# L-shaped association of serum 25-hydroxyvitamin D concentrations with cardiovascular and all-cause mortality in individuals with osteoarthritis: results from the NHANES database prospective cohort study

**DOI:** 10.1186/s12916-022-02510-1

**Published:** 2022-09-21

**Authors:** Qingqing Xiao, Bin Cai, Anwen Yin, Huanhuan Huo, Keke Lan, Guo Zhou, Linghong Shen, Ben He

**Affiliations:** 1 Department of Cardiology, Shanghai Chest Hospital, Shanghai Jiao Tong University School of Medicine, Shanghai, China; 2 Department of Rheumatology and Immunology, Shanghai Changzheng Hospital, Naval Medical University, Shanghai, China

**Keywords:** Osteoarthritis, 25-hydroxyvitamin D, Mortality, Cardiovascular diseases, Cancer mortality

## Abstract

**Background:**

The relationship between vitamin D status and mortality in patients with osteoarthritis (OA) is unknown. This study investigated the associations of serum 25-hydroxyvitamin D [25(OH)D] concentrations with all-cause and cause-specific mortality among American adults with OA.

**Methods:**

This study included 2556 adults with OA from the National Health and Nutrition Examination Survey (2001–2014). Death outcomes were ascertained by linkage to National Death Index (NDI) records through 31 December 2015. Cox proportional hazards model and two-piecewise Cox proportional hazards model were used to elucidate the nonlinear relationship between serum 25(OH)D concentrations and mortality in OA patients, and stratified analyses were performed to identify patients with higher mortality risk.

**Results:**

During 16,606 person-years of follow-up, 438 all-cause deaths occurred, including 74 cardiovascular disease (CVD)-related and 78 cancer deaths. After multivariable adjustment, lower serum 25(OH)D levels were significantly and nonlinearly associated with higher risks of all-cause and CVD mortality among participants with OA. Furthermore, we discovered L-shaped associations between serum 25(OH)D levels and all-cause and CVD mortality, with mortality plateauing at 54.40 nmol/L for all-cause mortality and 27.70 nmol/L for CVD mortality. Compared to participants with 25(OH)D levels below the inflection points, those with higher levels had a 2% lower risk for all-cause mortality (hazard ratio [HR] 0.98, 95% confidence interval [CI] 0.96–0.99) and 17% lower risk for CVD mortality (HR 0.83, 95% CI 0.72–0.95).

**Conclusions:**

Nonlinear associations of serum 25(OH)D levels with all-cause and CVD mortality were observed in American patients with OA. The thresholds of 27.70 and 54.40 nmol/L for CVD and all-cause mortality, respectively, may represent intervention targets for lowering the risk of premature death and cardiovascular disease, but this needs to be confirmed in large clinical trials.

## Background

Osteoarthritis (OA) is a complex chronic disease that currently affects an estimated 250 million people worldwide [1]. OA involves the spine and peripheral joints, resulting in severe disability [2, 3], and numerous studies have shown that individuals with OA have a higher risk of cardiovascular disease (CVD) and death than the general population [4–6]. Therefore, identifying modifiable factors to prevent OA complications and reduce mortality, particularly the risk of cardiovascular death, is critical.

25-hydroxyvitamin D [25(OH)D]—the primary storage form of vitamin D—is a kind of sterol and fat-soluble substance that promotes the absorption of calcium and phosphate [7]. Serum 25(OH)D deficiency is a common risk factor for a variety of diseases, including skeletal disease, CVD, hypertension, autoimmune disease, obesity, diabetes, chronic kidney disease, and depression [8–11]. In patients with OA, lower levels of serum 25(OH)D are associated with greater knee pain and increased progression of radiographic OA [12]. Although epidemiological studies have demonstrated that vitamin D deficiency is related to an increased risk of CVD and mortality in the general population [13], the relationships between vitamin D status and all-cause and cause-specific mortality in OA patients have not been reported and require further clarification. Furthermore, randomized clinical trials and meta-analyses have found no beneficial effects of vitamin D supplementation on overall mortality and CVD outcomes [13–15]. Because there is no agreement on optimal serum 25(OH)D concentrations in different populations, determining the optimal vitamin D concentration in OA patients is critical.

To fill these knowledge gaps, our primary objective was to prospectively investigate the association of serum 25(OH)D concentrations with all-cause and cause-specific mortality in a nationally representative sample of American adult patients with OA. The secondary goal was to recommend vitamin D supplementation in patients with OA by determining optimal serum 25(OH)D concentrations.

## Methods

### Study design and population

**Fig. 1:**
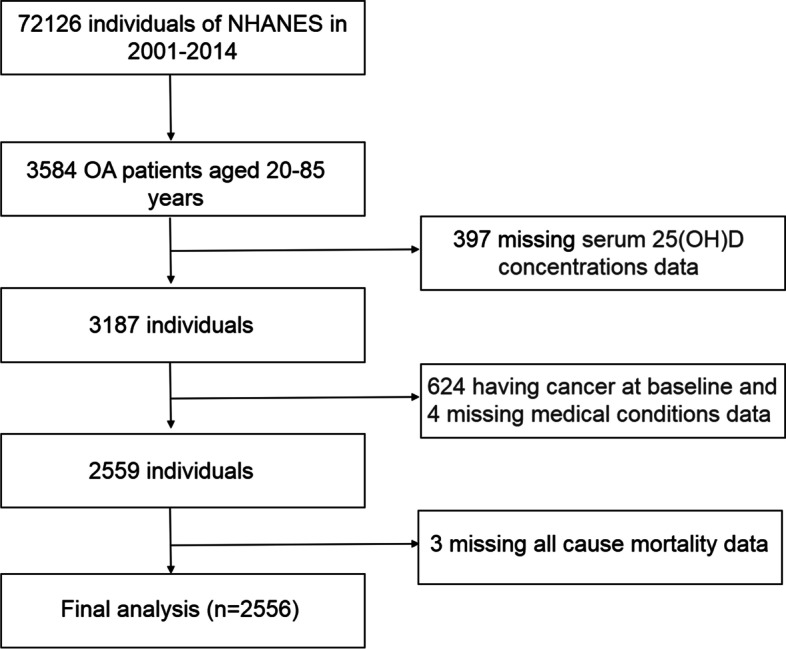
Flow chart of study participants

National Health and Nutrition Examination Survey (NHANES) was a nationally representative survey of the civilian, noninstitutionalized US population, which was conducted by the National Center for Health Statistics (NCHS) of the Centers for Disease Control and Prevention (CDC) [16]. The protocols of NHANES were approved by the Research Ethics Review Board of NCHS [17]. NHANES has obtained informed written consent from all participants. The datasets generated and analyzed in the current study are available at NHANES website (https://www.cdc.gov/nchs/nhanes/index.htm). We downloaded seven cycles of NHANES from 2001 to 2014 (2001–2002, 2003–2004, 2005–2006, 2007–2008, 2009–2010, 2011–2012, and 2013–2014). The data of each cycle contain five parts: demographics data, dietary data, examination data, laboratory data, and questionnaire data. The downloaded data were visualized and analyzed using the statistical package R (R 3.4.3) and EmpowerStats 2.0. In this study, a total of 72,126 participants were surveyed. Patients with OA were found in self-reported personal interview data on a variety of health conditions (*n* = 3584, aged 20 to 85 years old). A total of 2556 participants were included in the final analysis, after excluding those with missing serum 25(OH)D concentrations (*n* = 397), cancer at baseline (*n* = 624), missing medical conditions data (*n* = 4), and all-cause mortality data (*n* = 3) (Fig. 1).

### Measurement of serum 25(OH)D concentrations

The serum 25(OH)D concentrations were measured by DiaSorin RIA kit (Stillwater MN) in NHANES 2001–2006 and by a standardized liquid chromatography-tandem mass spectrometry (LC-MS/MS) method in NHANES 2007–2014. To enable researchers to use and analyze 25(OH)D concentrations, RIA measurements of 25(OH)D concentration were converted to equivalent 25(OH)D measurements in the standardized LC-MS/MS method using regression methods to adjust for assay drifts. The analysis was performed using the LC-MS/MS method following the CDC recommendations [18].

### Determination of mortality outcomes

To determine mortality status in the follow-up population, we used the NHANES public-use linked mortality file as of December 31, 2015, which was correlated with NCHS with the National Death Index (NDI) through a probability matching algorithm. Furthermore, disease-specific death was determined using the International Statistical Classification of Diseases, 10th Revision (ICD-10), and the NCHS classified heart diseases (054-064), malignant neoplasms (019-043), and all other causes (010) [19].

### Assessment of covariates

NHANES personnel used questionnaires during home interviews to collect information on age, gender, race/ethnicity, education levels, marital status, family income, smoking status, alcohol consumption, and disease status using questionnaires (disease duration of OA, history of hypertension or diabetes). At a Mobile Examination Center, participants’ height, weight, waist circumference, and body mass index (BMI, kg/m^2^) were measured.

Furthermore, race/ethnicity was classified as Mexican American, other Hispanic, non-Hispanic White, non-Hispanic Black, or other race; education levels were categorized as less than high school, high school or equivalent, college, or above; family income was classified as low income, middle income, and high income, based on the family poverty income ratio (PIR, < 1.30, 1.30–3.49, ≥ 3.50), respectively; alcohol consumption was classified by whether ≥ 4 drinks/day, smoking status was categorized as daily, sometimes, and not at all.

Serum specimens were collected as part of the 2001–2014 NHANES laboratory examination component, and rigorous procedures were used throughout the blood collection and analysis process [20]. At baseline, plasma glycohemoglobin (%), glucose (mg/dL), cholesterol (mg/dL), direct HDL-cholesterol (mg/dL), LDL-cholesterol (mg/dL), triglycerides (mg/dL), uric acid (mg/dL), and C-reactive protein (CRP, mg/dL) levels were measured. Furthermore, we computed the estimated glomerular filtration rate (eGFR) using the Chronic Kidney Disease Epidemiology Collaborative equation to assess the participants’ kidney function [21].

### Statistical analyses

The data in this study were statistically analyzed in accordance with CDC guidelines [22]. Serum 25(OH)D levels were categorized according to the Endocrine Society Clinical Practice guidelines [23], as follows: <25.00 nmol/L, indicative of severe vitamin D deficiency; 25.00–49.99 nmol/L, indicative of vitamin D deficiency; 50.00–74.99 nmol/L, indicative of vitamin D insufficiency; ≥75.00 nmol/L, indicative of vitamin D sufficiency. The *χ*^2^ test (categorical variables) and linear regression models (continuous variables) were used to calculate differences between the four 25(OH)D groups, with data expressed as numbers with percentages for categorical variables and as means with standard deviations for continuous variables. Three Cox regression models were developed to investigate the relationship between serum 25(OH)D concentrations and mortality: Model 1 (unadjusted); Model 2 was adjusted for gender, age, race/ethnicity, and survey cycle; Model 3 was adjusted for gender, age, race/ethnicity, survey cycle, education level, PIR, BMI, smoking status, alcohol consumption, OA disease duration, hypertension, and diabetes.

To investigate the relationship between serum 25(OH)D and mortality in patients with OA, Cox proportional risk regression models were developed using generalized additive models and smoothed curve fitting (penalized spline method). If the relationship was nonlinear, we used a recursive algorithm to calculate the inflection points between 25 (OH) D and all-cause and CVD mortality, respectively, and a two-segment Cox proportional risk model was used on both sides of the inflection point to investigate the association between serum 25(OH)D and the risk of all-cause mortality. Stratified analyses were conducted based on gender, age (< 60 years old or ≥ 60 years old), race/ethnicity (Whites or non-Whites), hypertension, diabetes, and BMI (< 25.00 or ≥ 25.00). All analyses were carried out with R 3.4.3 and EmpowerStats 2.0, and a two-tailed *P* < 0.05 was considered statistically significant.

## Results

### Baseline characteristics of study participants

In this study, the analysis dataset consisted of data from 2556 patients with OA (mean age: 63.01 ± 13.79 years; 35.88% males). The weighted mean concentration of serum 25(OH)D was 70.80 ± 27.00 nmol/L; 26.95% had deficient vitamin D (< 50.00 nmol/L), and 64.32% had insufficient vitamin D (< 75.00 nmol/L). The baseline characteristics of the research population according to serum 25(OH)D status were displayed in Table 1. Participants who had higher serum 25(OH)D levels were more likely to be older and non-Hispanic White, had higher education levels and family income, were less likely to be obese and have alcohol consumption (*P* < 0.01). As shown in Table 2, when cardiometabolic biomarkers were compared based on serum 25(OH)D levels, higher serum 25(OH)D levels were significantly associated with lower levels of glycohemoglobin, glucose, eGFR, uric acid, and CRP at baseline (*P* < 0.01).

**Table 1:** Baseline characteristics of participants with OA according to serum 25(OH)D concentrations

	Serum 25(OH)D concentrations (nmol/L)					***P***-value
	Total	< 25.00	25.00–49.99	50.00–74.99	≥ 75.00	
***N*(%)**	2556 (100.00)	100 (3.91)	589 (23.04)	955 (37.36)	912 (35.68)	
**Age (years)**	63.01 ± 13.79	60.12 ± 14.26	61.84 ± 14.06	62.59 ± 13.91	64.52 ± 13.30	<0.01
**Gender**						<0.01
Male (%)	917 (35.88)	25 (25.00)	207 (35.14)	394 (41.26)	291 (31.91)	
Female (%)	1639 (64.12)	75 (75.00)	382 (64.86)	561 (58.74)	621 (68.09)	
**Race/ethnicity**						<0.01
Mexican American (%)	238 (9.31)	17 (17.00)	85 (14.43)	88 (9.21)	48 (5.26)	
Other Hispanic (%)	157 (6.14)	5 (5.00)	44 (7.47)	70 (7.33)	38 (4.17)	
Non-Hispanic White (%)	1667 (65.22)	29 (29.00)	285 (48.39)	631 (66.07)	722 (79.17)	
Non-Hispanic Black (%)	376 (14.71)	44 (44.00)	143 (24.28)	116 (12.15)	73 (8.00)	
Other race—including multi-racial (%)	118 (4.62)	5 (5.00)	32 (5.43)	50 (5.24)	31 (3.40)	
**Education (%)**						<0.01
Less than high school	613 (23.99)	35 (35.00)	180 (30.56)	219 (22.93)	179 (19.63)	
High school or equivalent	620 (24.27)	24 (24.00)	131 (22.24)	226 (23.66)	239 (26.21)	
College or above	1322 (51.74)	41 (41.00)	278 (47.20)	510 (53.40)	493 (54.06)	
Not recorded	1 (0.04)	0 (0.00)	0 (0.00)	0 (0.00)	1 (0.11)	
**Smoking status (%)**						<0.01
Every day	375 (14.67)	23 (23.00)	110 (18.68)	133 (13.93)	109 (11.95)	
Some day	53 (2.07)	3 (3.00)	16 (2.72)	18 (1.88)	16 (1.75)	
Not at all	903 (35.33)	26 (26.00)	187 (31.75)	358 (37.49)	332 (36.40)	
Not recorded	1225 (47.93)	48 (48.00)	276 (46.86)	446 (46.70)	455 (49.89)	
**Alcoholic ≥ 4 drinks/day (%)**						0.03
Yes	303 (11.85)	13 (13.00)	75 (12.73)	131 (13.72)	84 (9.21)	
No	1488 (58.22)	52 (52.00)	327 (55.52)	547 (57.28)	562 (61.62)	
Not recorded	765 (29.93)	35 (35.00)	187 (31.75)	277 (29.01)	266 (29.17)	
**Family poverty income ratio**	2.71 ± 1.62	2.29 ± 1.53	2.39 ± 1.56	2.77 ± 1.62	2.89 ± 1.63	<0.01
**Weight (kg)**	83.90 ± 22.82	97.08 ± 31.80	88.04 ± 24.17	84.76 ± 22.53	78.98 ± 19.74	<0.01
**BMI (kg/m**^**2**^**)**	30.74 ± 7.60	36.55 ± 10.99	32.49 ± 7.97	30.62 ± 7.18	29.13 ± 6.78	<0.01
**Waist circumference (cm)**	103.57 ± 15.97	113.69 ± 20.71	107.40 ± 15.77	103.54 ± 15.56	100.24 ± 15.05	<0.01
**Duration of OA (%)**						0.20
Less than 5 years	745 (29.15)	26 (26.00)	163 (27.67)	300 (31.41)	256 (28.07)	
5–10 years	486 (19.01)	22 (22.00)	107 (18.17)	192 (20.10)	165 (18.09)	
10-20 years	712 (27.86)	22 (22.00)	162 (27.50)	266 (27.85)	262 (28.73)	
More than 20 years	597 (23.36)	29 (29.00)	151 (25.64)	193 (20.21)	224 (24.56)	
No Recorded	16 (0.63)	1 (1.00)	6 (1.02)	4 (0.42)	5 (0.55)	
**Hypertension (%)**	1462 (57.20)	67 (67.00)	346 (58.74)	518 (54.24)	531 (58.22)	<0.01
**Diabetes (%)**	455 (17.80)	30 (30.00)	144 (24.45)	162 (16.96)	119 (13.05)	<0.01

Data are presented as mean ± SD or *n* (%)

**Table 2:** Baseline levels of cardiometabolic markers according to serum 25(OH)D concentrations among participants with OA

	Serum 25(OH)D concentrations (nmol/L)				***P***-value
	< 25.00	25.00–49.99	50.00–74.99	≥ 75.00	
**Glycohemoglobin (%)**	6.11 ± 1.00	6.01 ± 1.17	5.81 ± 0.88	5.76 ± 0.72	<0.01
**Glucose, refrigerated serum (mg/dL)**	107.38 ± 37.24	111.62 ± 48.65	103.66 ± 33.25	102.25 ± 28.10	<0.01
**Cholesterol (mg/dL)**	199.24 ± 39.41	201.03 ± 45.62	199.61 ± 42.95	201.46 ± 42.75	0.76
**Direct HDL-cholesterol (mg/dL)**	54.96 ± 14.38	53.02 ± 16.13	53.15 ± 15.54	57.15 ± 16.90	<0.01
**LDL-cholesterol (mg/dL)**	111.00 ± 32.59	117.08 ± 37.38	116.48 ± 36.58	114.50 ± 36.59	0.48
**Triglycerides (mg/dL)**	153.18 ± 98.98	159.22 ± 117.47	159.46 ± 110.97	159.37 ± 108.93	0.81
**eGFR (mL/min/1.73 m**^**2**^**)**	81.14 ± 27.80	80.81 ± 25.29	77.11 ± 20.91	73.37 ± 20.86	<0.01
**Uric acid (mg/dL)**	6.15 ± 1.70	5.64 ± 1.45	5.58 ± 1.47	5.48 ± 1.45	<0.01
**C-reactive protein (mg/dL)**	0.84 ± 1.39	0.55 ± 0.66	0.50 ± 0.80	0.49 ± 0.96	<0.01

Mean ± SD for continuous variables: *P*-value was calculated by weighted linear regression model

### Relationships of 25(OH)D concentration with mortality

During 16,606 person-years of follow-up, 438 all-cause deaths occurred, including 74 CVD-related deaths and 78 cancer deaths (Table 3). We designed 3 Cox regression models to investigate the independent role of serum 25(OH)D levels in mortality. After multivariate adjustment including age, sex, race/ethnicity, survey cycle, education level, PIR, BMI, smoking status, alcohol consumption, disease duration of OA, history of hypertension or diabetes (Model 3), the multivariate-adjusted hazard ratios (HRs) and 95% confidence intervals (CIs) from lowest to highest serum 25(OH)D categories (< 25.00, 25.00–49.99, 50.00–74.99, and ≥ 75.00 nmol/L) were 1.00 (reference), 0.53 (0.29, 0.96), 0.43 (0.24, 0.77), and 0.44 (0.24, 0.81), respectively, for all-cause mortality (*P* trend = 0.02); 1.00 (reference), 0.16 (0.04, 0.60), 0.19 (0.05, 0.68), and 0.18 (0.05, 0.68), respectively, for CVD mortality (*P* trend = 0.41); and 1.00 (reference), 0.65 (0.17, 2.41), 0.63 (0.17, 2.35), and 0.47 (0.12, 1.87), respectively, for cancer mortality (*P* trend = 0.40). We also discovered that OA with higher levels of serum 25(OH)D (≥ 25.00 nmol/L) had lower all-cause and CVD mortality than those with serum 25(OH)D < 25.00 nmol/L, but no considerable difference in cancer mortality.

**Table 3:** HRs (95% CIs) for mortality according to serum 25(OH)D concentrations among participants with OA

	Serum 25(OH)D concentrations (nmol/L)				***P*** trend
	< 25.00	25.00–49.99	50.00–74.99	≥ 75.00	
**All-cause mortality**					
Number of deaths (%)	24 (24.00)	126 (21.39)	173 (18.12)	115 (12.61)	
Model 1 HR (95% CI) *P*-value	1.00	0.78 (0.50, 1.20) 0.25	0.65 (0.42, 0.99) <0.05	0.59 (0.38, 0.92) 0.02	<0.01
Model 2 HR (95% CI) *P*-value	1.00	0.59 (0.38, 0.92) 0.02	0.42 (0.27, 0.66) <0.01	0.38 (0.24, 0.60) <0.01	<0.01
Model 3 HR (95% CI) *P*-value	1.00	0.53 (0.29, 0.96) 0.04	0.43 (0.24, 0.77) <0.01	0.44 (0.24, 0.81) <0.01	0.02
**CVD mortality**					
Number of deaths (%)	4 (4.00)	20 (3.40)	31 (3.25)	19 (2.08)	
Model 1 HR (95% CI) *P*-value	1.00	0.76 (0.26, 2.23) 0.62	0.73 (0.26, 2.06) 0.55	0.57 (0.19, 1.66) 0.30	0.09
Model 2 HR (95% CI) *P*-value	1.00	0.62 (0.21, 1.87) 0.40	0.47 (0.16, 1.40) 0.18	0.37 (0.12, 1.15) 0.08	0.57
Model 3 HR (95% CI) *P*-value	1.00	0.16 (0.04, 0.60) <0.01	0.19 (0.05, 0.68) 0.01	0.18 (0.05, 0.68) 0.01	0.41
**Cancer mortality**					
Number of deaths (%)	3 (3.00)	20 (3.40)	34 (3.56)	21 (2.30)	
Model 1 HR (95% CI) *P*-value	1.00	0.98 (0.29, 3.31) 0.98	1.00 (0.31, 3.26) 1.00	0.86 (0.26, 2.89) 0.81	0.24
Model 2 HR (95% CI) *P*-value	1.00	0.85 (0.25, 2.91) 0.80	0.81 (0.24, 2.73) 0.73	0.64 (0.18, 2.26) 0.49	0.10
Model 3 HR (95% CI) *P*-value	1.00	0.65 (0.17, 2.41) 0.52	0.63 (0.17, 2.35) 0.49	0.47 (0.12, 1.87) 0.28	0.40

Model 1: Non-adjusted

Model 2: Adjusted for age, sex, race/ethnicity, survey cycle

Model 3: Adjusted for age, sex, race/ethnicity, survey cycle, education level, PIR, BMI, smoking status, alcohol consumption, disease duration of OA, and history of hypertension or diabetes

### The detection of nonlinear relationships

**Fig. 2:**
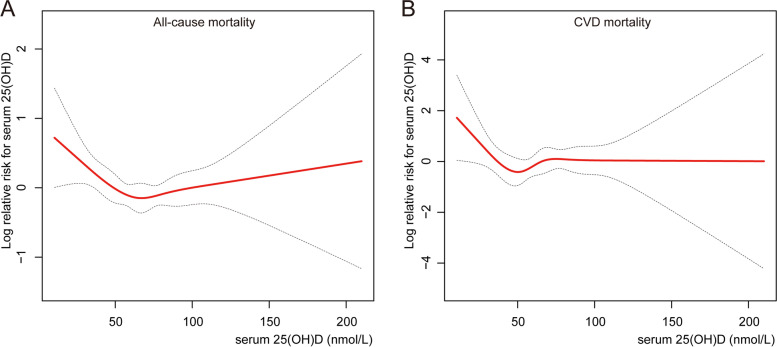
Association between 25(OH)D concentration and all-cause (**A**) and CVD mortality (**B**) in patients with OA. Adjusted for age, sex, race/ethnicity, survey cycle, education level, family poverty income ratio, BMI, smoking status, alcohol consumption, disease duration of OA, and history of hypertension or diabetes. The solid and dotted lines represent the estimated values and their corresponding 95% CIs, respectively

By generalized additive models and smoothed curve fitting (penalized spline method), we discovered the L-shaped associations between serum 25(OH)D concentrations and all-cause (Fig. 2A) and CVD mortality (Fig. 2B). We then combined a Cox proportional hazards model with a two-piecewise Cox proportional hazards model to investigate the nonlinear relationship between serum 25(OH)D levels and all-cause and CVD mortality in patients with OA (both *P* for log-likelihood ratio < 0.05) (Table 4). We discovered that the inflection points for all-cause mortality were 54.40 nmol/L and 27.70 nmol/L for CVD mortality. When serum 25(OH)D concentrations were less than 54.40 nmol/L or 27.70 nmol/L, a 1-unit decrease in the 25(OH)D level was associated with a 2% and a 17% greater adjusted HR of all-cause and CVD mortality, respectively (HR 0.98; 95% CI 0.96, 0.99 and HR 0.83; 95% CI 0.72, 0.95, respectively). When serum 25(OH)D concentrations exceeded 54.40 nmol/L or 27.70 nmol/L, respectively, there was no association with all-cause or CVD mortality (HR 1.00; 95% CI 1.00, 1.01 and HR 1.00; 95% CI 0.99, 1.02, respectively).

**Table 4:** Threshold effect analysis of serum 25(OH)D concentrations on all-cause and CVD mortality in OA patients

	Adjusted HR (95% CI), ***P***-value
**All-cause mortality**	
Fitting by the standard linear model	1.00 (0.99, 1.00) 0.31
Fitting by the two-piecewise linear model	
Inflection point	54.40 nmol/L
25(OH)D concentrations < 54.40 nmol/L	0.98 (0.96, 0.99) < 0.01
25(OH)D concentrations ≥ 54.40 nmol/L	1.00 (1.00, 1.01) 0.40
*P* for Log-likelihood ratio	0.02
**CVD mortality**	
Fitting by the standard linear model	1.00 (0.99, 1.01) 0.84
Fitting by the two-piecewise linear model	
Inflection point	27.70 nmol/L
25(OH)D concentrations < 27.70 nmol/L	0.83 (0.72, 0.95) < 0.01
25(OH)D concentrations ≥ 27.70 nmol/L	1.00 (0.99, 1.02) 0.56
*P* for log-likelihood ratio	0.02

Adjusted for age, sex, race/ethnicity, survey cycle, education level, PIR, BMI, smoking status, alcohol consumption, disease duration of OA, and history of hypertension or diabetes

### Stratified analyses

**Fig. 3:**
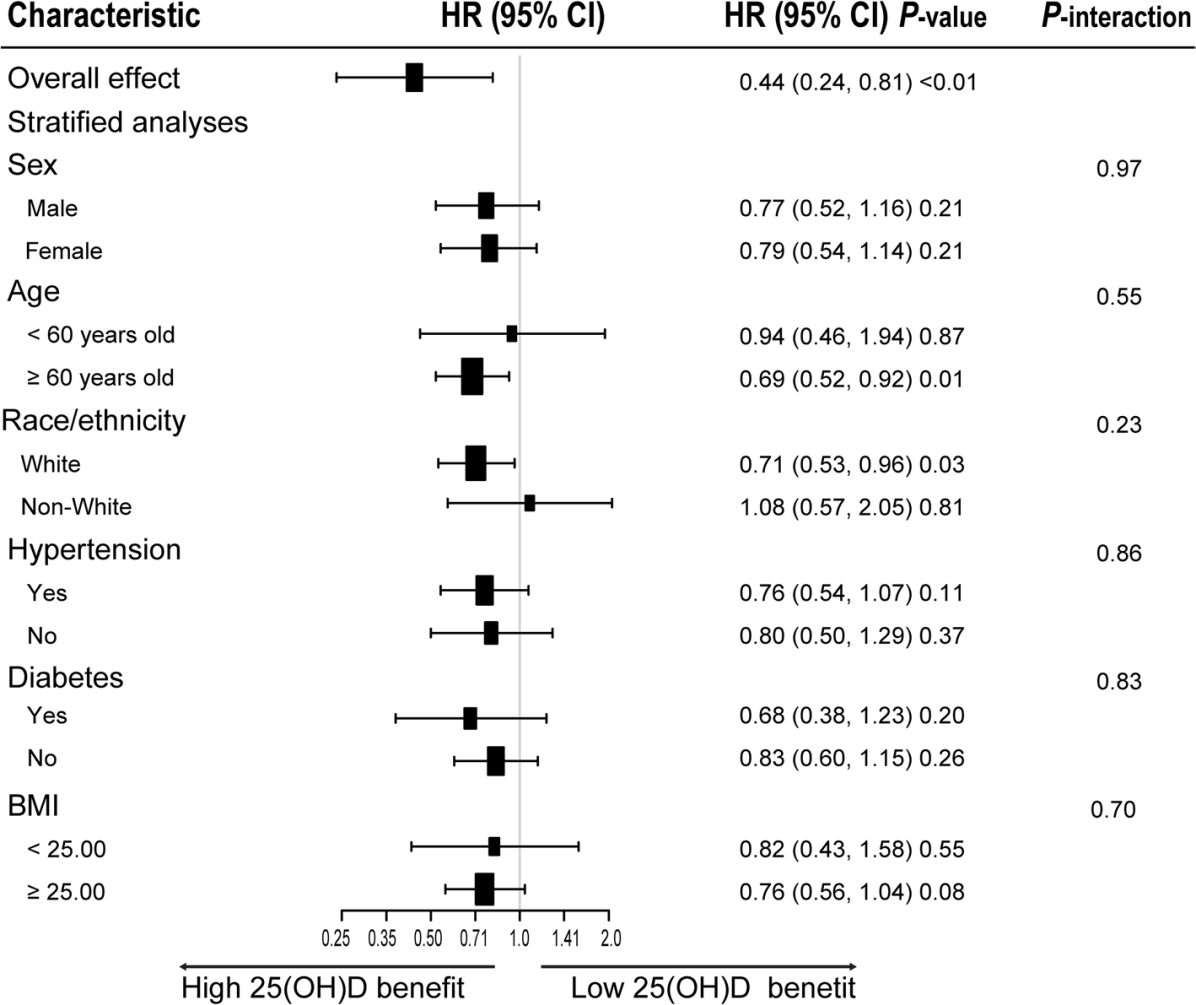
Forest plots of stratified analyses of 25(OH)D and all-cause mortality. Age, sex, race/ethnicity, survey cycle, education level, PIR, BMI, smoking status, alcohol consumption, disease duration of OA, and history of hypertension or diabetes were all adjusted except the variable itself

The advantage of higher serum 25(OH)D concentrations (≥ 54.40 nmol/L) versus lower serum 25(OH)D concentration (< 54.40 nmol/L) on survival of OA patients was similar across a wide range of subgroups stratified by age, sex, race, BMI, history of hypertension, and history of diabetes (Fig. 3). There was no significant interaction between serum 25(OH)D concentration and stratified variables. Meanwhile, our findings showed that a stronger inverse association between vitamin D status and all-cause mortality was found in older (≥ 60 years old) and White patients with OA, although the interaction test was not distinct.

## Discussion

To our knowledge, this is the first prospective study with a relatively large sample size to examine the link between serum 25(OH)D levels and all-cause and CVD mortality in OA patients. We discovered L-shaped relationships between serum 25(OH)D levels and all-cause and CVD mortality in OA patients in this cohort study, implying that lower serum 25(OH)D levels were significantly associated with higher all-cause and CVD mortality within a certain range. These findings could lead to clinical and dietary recommendations for lowering all-cause and CVD mortality in OA patients.

Similar to other studies [24, 25], our data revealed that 64.32% of OA patients with serum 25(OH)D insufficiency, indicating that vitamin D deficiency was widespread in OA patients. In our research, OA patients with serum 25(OH)D < 25.00 nmol/L had higher all-cause and CVD mortality compared with those with serum 25(OH)D ≥ 25.00 nmol/L, and the L-shaped relationships were seen between serum 25(OH)D concentrations and the all-cause and CVD mortality, which was similar to the large group investigation from the general population in the UK [26]. This study finding was consistent with previous reports that indicated low serum 25(OH)D concentration was associated with an increased risk of all-cause and CVD death [26–31]. The association was generally nonlinear, with mortality decreasing with an increase in 25(OH)D up to a certain point, beyond which mortality did not decline further. Concentration of 25(OH)D < 54.40 nmol/L in OA patients increased the risk of premature death, and 25(OH)D concentration < 27.70 nmol/L had a significant risk of CVD death. Fan et.al reported that the concentration of 25(OH)D < 60.00 nmol/L could increase the risk of CVD death and premature death [26], which seemed to indicate that the demand for vitamin D in different populations is inconsistent. However, there is no clear benefit of vitamin D supplementation for all-cause and CVD mortality in the general population [14, 15]. Because there was no further reduction in mortality when serum vitamin D concentration reached a certain threshold, L-shaped relationships between serum 25(OH)D concentrations and all-cause and CVD mortality could partially explain this phenomenon.

It is well known that vitamin D played an essential role in bone health and the pathophysiology of several chronic diseases, but the optimal concentration of serum 25(OH)D is still controversial. According to the Endocrinology Society and the International Osteoporosis Foundation, the optimal concentration of serum 25(OH)D in the general adult population should be at least 75.00 nmol/L for better health outcomes (including mortality) [23, 32, 33]. However, the American Institute of medicine believed that based on its key role in bone health, 50.00 nmol/L was sufficient [34]. A meta-analysis of 14 prospective cohort studies demonstrated that the risk of death reduced nonlinearly with the increase of serum 25(OH)D, and the optimal concentration was about 75.00–87.50 nmol/L [35]. The results were inconclusive due to differences in sample size, follow-up time, and target population. In our current study, we found thresholds for CVD mortality of 27.70 nmol/L and all-cause mortality of 54.40 nmol/L in the OA population. Whether serum 25(OH)D concentration ≥ 54.40 nmol/L can decrease the overall premature death and cardiovascular risk of the OA population needs to be confirmed in future clinical trials.

There is controversy regarding the relationship between vitamin D and cancer mortality [36]. A meta-analysis of 12 prospective cohorts reported that higher baseline serum 25(OH)D levels were associated with a lower risk of cancer mortality [36, 37]; however, other prospective studies revealed no association between serum vitamin D levels and cancer mortality [38, 39]. There was no significant relationship between serum 25(OH)D levels and cancer mortality in OA patients in our study. Larger prospective studies are needed to investigate the link between vitamin D and cancer mortality.

The identification of populations at higher risk of all-cause mortality in patients with OA might have significant public health implications [4]. Based on the stratified analysis, we observed the advantage in all-cause mortality from higher serum 25(OH)D (≥ 54.40 nmol/L) in patients with OA, particularly in elderly patients (≥ 60 years old) and White patients. Previous studies demonstrated that the prevalence of severe vitamin D deficiency was 15- to 20-fold higher in African Americans compared to European Americans [40]. Our study also revealed that 44.00% of non-Hispanic Blacks with serum 25(OH)D below 25.00 nmol/L in the OA patients, however, vitamin D deficiency appeared to be more harmful in Whites patients compared with non-Whites patients. A study of the White population in the United States found that vitamin D deficiency was associated with an increased risk of stroke death, but not with Blacks [41]. One possible explanation is that Blacks have adapted to be relatively resistant to the negative effects of 25(OH)D deficiency [42]. Furthermore, similar to previous research [43], our study supported a higher risk of death in the elderly population with low vitamin D. As a result, when managing OA patients, we should consider monitoring vitamin D levels during clinical review and supplementing accordingly, particularly in an elderly patient (≥ 60 years old), and White patients.

Several possible mechanisms could explain the link between lower 25 (OH) D levels and an increased risk of death. Low vitamin D levels were associated with a variety of diseases which contributed to deaths, such as obesity and dyslipidemia, hypertension, and diabetes mellitus [44, 45]. Low levels of vitamin D were also associated with CVD, such as coronary artery calcification, increased carotid intima-media thickness, and elevated serum triglyceride levels [46–48]. Secondary hyperparathyroidism associated with low level of vitamin D is reported to increase the risk of cardiovascular mortality. Secondary hyperparathyroidism activated renin-angiotensin-aldosterone system, elevated blood pressure, activated systemic inflammatory response and macrophage inflammatory response, promoted cholesterol accumulation in the vascular wall, and aggravated atherosclerosis [49–51]. In the present study, we further discovered that the lower 25(OH)D levels were primarily associated with increased CVD mortality. This might be because participants with lower 25(OH)D levels seemed to have poor profiles (high cardiovascular risk, comorbidity, unhealthy lifestyle, and low education). Therefore, future studies are needed to clarify the potential mechanism of vitamin D in the prevention of all-cause and CVD mortality.

There are some benefits in this study. First, we used a nationally representative sample of adults with OA in the United States, which had a relatively large sample size and helped generalize our results. Furthermore, the number of cardiovascular events and deaths in long-term follow-ups provided sufficient strength for the analysis in the present study. Second, by adjusting for socioeconomic status, diet and lifestyle factors, comorbidity, and other potential confounding factors, we could improve the effectiveness of the conclusion. Finally, the serum 25 (OH) D concentrations in the NHANES database were measured using a standard method, ensuring the data analysis reliability.

There are some limitations to our study. First, since this was an observational study, cause and impact could not be determined. Second, although a single measurement of serum 25 (OH) D was a reasonable proxy for evaluating vitamin D status [52], the current study measured serum 25 (OH) D concentrations only once at baseline, which would underestimate the correlation of interest [53]. Third, our study, like other observational studies, could not rule out residual or unknown confounding or accidental confounding effects due to measurement errors and unmeasured variables (i.e., psychosocial stress or genetic susceptibility).

## Conclusions

After multivariable adjustment, lower serum 25(OH)D levels were significantly and nonlinearly associated with higher risks of all-cause and CVD mortality in American adults with OA. We further discovered L-shaped associations between serum 25(OH)D levels and all-cause and CVD mortality plateaued at 54.40 nmol/L for all-cause mortality and at 27.70 nmol/L for CVD mortality. The thresholds of 27.70 and 54.40 nmol/L might represent intervention targets for lowering the risk of premature death and cardiovascular disease. These results highlight the potential advantages of monitoring and evaluating vitamin D status in the prevention of CVD and mortality among adults with OA.

## Data Availability

The datasets generated and analyzed in the current study are available at NHANES website: https://www.cdc.gov/nchs/nhanes/index.htm.

## Abbreviations

OA: Osteoarthritis
CVD: Cardiovascular disease
25(OH)D: 25-Hydroxyvitamin D
95% CI: 95% confidence interval
HR: Hazard ratio
PIR: Family poverty income ratio
NHANES: National Health and Nutrition Examination Survey
NCHS: National Center for Health Statistics
CDC: Centers for Disease Control and Prevention
NDI: National death index
